# The Combined Effects of Per- and Polyfluoroalkyl Substances, Metals, and Behavioral and Social Factors on Depressive Symptoms

**DOI:** 10.3390/medsci13020069

**Published:** 2025-06-01

**Authors:** Olamide Ogundare, Emmanuel Obeng-Gyasi

**Affiliations:** 1 Department of Built Environment, North Carolina A&T State University, Greensboro, NC 27411, USA; 2 Environmental Health and Disease Laboratory, North Carolina A&T State University, Greensboro, NC 27411, USA

**Keywords:** Bayesian, mixtures, epidemiology, health

## Abstract

*Background:* This study investigates the combined effects of PFAS metals (PFOA and PFOS), heavy metals (lead, cadmium, and mercury), behavioral factors (smoking and alcohol consumption), and social factors (income and education) on depressive symptoms. *Methods:* Using cross-sectional data from the National Health and Nutrition Examination Survey (NHANES 2017–2018), blood samples were analyzed to determine the exposure levels of PFOA, PFOS, lead, cadmium, and mercury, and self-reported behavioral and social factors were evaluated in relation to PHQ-9 scores among 181 adults. *Results:* Education was associated with lower odds of depressive symptoms (OR = 0.68, 95% CI: 0.43–1.07). Although the result was not statistically significant, the estimate suggested a potential protective effect that warranted further investigation. Bayesian Kernel Machine Regression demonstrated that heavy metals collectively had the strongest evidence for influencing depression (group PIP = 0.6508), followed by socioeconomic factors (group PIP = 0.642). Bivariate exposure–response analyses revealed complex interaction patterns whereby exposure effects varied substantially depending on co-exposure contexts. *Conclusions:* These findings highlight that depressive symptoms are shaped by complex interplays between environmental contaminants, behavior, and social determinants, underscoring the importance of mixture-based approaches in environmental mental health research and the need for integrated interventions addressing both environmental and social factors.

## 1. Introduction

Over the last two decades, depression has become a major global public health concern, with a pronounced impact on low- and middle-income countries (LMICs) [1]. According to the assessment report on global disease burden by the World Health Organization (WHO), depression was the second highest burden and disability-causing disease among all diseases in 2020, and it is expected to become the world’s largest disease burden by 2030 [2]. Depressive disorders encompass a range of conditions marked by persistent low mood, reduced interest and pleasure in activities, decreased energy, negative self-perception, cognitive sluggishness, and disturbances in sleep, appetite, and psychomotor activity. The most severe form, major depressive disorder, is a leading cause of global disability and a significant risk factor for suicide [3]. Around 350 million people of all ages worldwide suffer from depression, which can negatively impact patients’ physical and mental health, quality of life, disease outcomes, and hospital-seeking behavior [4].

There is growing evidence of the role of environmental exposures in mental health. Mental disorders or depressive symptoms have complex, multifaceted, and interrelated environmental origins [5]. Exposure to pollution, noise, and other environmental stressors can affect mental well-being. Poverty, unemployment, and lack of access to education and healthcare can contribute to the development of psychiatric disorders. With increasing environmental pollution, many pollutants may impact human disease [6]. Per- and polyfluoroalkyl substances (PFASs) constitute a class of synthetic organic compounds characterized by the presence of carbon–fluorine bonds, which are among the strongest in organic chemistry. Owing to their remarkable physicochemical stability, PFASs have been widely used in industrial applications and consumer products since the 1940s. These compounds exhibit high resistance to thermal degradation, photolysis, chemical reactivity, and biotransformation, making them persistent in both environmental and biological systems [7]. Due to their excellent chemical stability, especially thermal stability, PFASs are widely used in industrial manufacturing, the electronics industry, food packaging, and personal care products [8].

Numerous studies have investigated the relationship between exposure to per- and polyfluoroalkyl substances (PFASs) and depressive symptoms in adult populations, as well as their implications for maternal health outcomes [9,10,11]. A review examining the metabolic perturbations associated with both PFAS exposure and perinatal/antenatal depression in pregnant women identified potential metabolic pathways that may mediate this relationship. The review highlighted several key pathways, including fatty acid and amino acid metabolism, which appear to be common to both PFAS exposure and perinatal depression. These metabolic disruptions may contribute to adverse pregnancy outcomes and increased susceptibility to mood disorders during the perinatal period [12]. In parallel, exposure to heavy metals such as lead, cadmium, and mercury has been independently associated with adverse effects on mental health, including cognitive and affective dysfunctions [13,14,15,16]. Despite growing evidence on the individual impacts of PFASs and metals on mental health, there remains a critical need to examine the effects of co-exposure to these environmental pollutants on depressive symptoms. Assessing joint effects poses methodological challenges due to the high degree of correlation often observed among environmental toxicants, which complicates the estimation of both independent and interactive effects. Moreover, pollutant interactions may exhibit non-linearity and complex dependency structures, including potential synergistic or antagonistic relationships. As such, elucidating these interactions necessitates the application of advanced statistical and computational modeling approaches capable of capturing the multidimensional and dynamic nature of multiple concurrent exposures [17].

Smoking and alcohol are significant behavioral factors linked to depression. Studies have shown that smoking is associated with a higher risk of developing depression. This relationship is dose-dependent, meaning the more a person smokes, the greater their risk of depression [18]. While moderate alcohol (up to 14 standard drinks/week for men and up to 7 standard drinks/week for women) consumption might not significantly impact mental health, heavy drinking (above the standard level) is strongly associated with an increased risk of depression [19]. Income and education are significant social determinants that also profoundly affect mental health outcomes. Higher income levels provide better access to resources such as healthcare, nutritious food, and safe housing, all of which are crucial for maintaining good mental health. Lower income is often associated with financial instability and stress, which can contribute to the development of mental health issues like depression and anxiety [20]. Higher levels of education are linked to better health literacy, which includes understanding how to maintain mental health and access to mental health services. Education improves employment opportunities, which can lead to better job security and financial stability, both of which are protective factors against mental health issues [21]. The combined effects of higher income and education can create a positive feedback loop, enhancing overall well-being and reducing the risk of mental health disorders [22].

There is a complex interplay between environmental, behavioral, and social factors in the sense that the combined impact of environmental exposures, behavioral factors, and social determinants can lead to more severe health outcomes [23]. For instance, individuals with low socioeconomic status who smoke and live in polluted areas are likely to be at a significantly higher risk of developing health issues compared to those who do not have these combined risk factors. Research has established that children from economically disadvantaged communities face significantly higher exposure to environmental neurotoxins, including lead and atmospheric pollutants, potentially intensifying the neurological and psychological impacts of childhood social stressors [24]. Studies exploring the intersection of environmental exposures and mental health reveal that individuals experiencing economic constraints are more susceptible to neurotoxic impacts, with chronic stress and restricted healthcare access potentially amplifying their vulnerability [25]. Existing research often focuses on isolated exposures, examining the effects of a single type of exposure, whether environmental, behavioral, or social, on health outcomes. In the real world, people are typically exposed to multiple factors simultaneously. Studies that focus on isolated exposures do not account for the combined effects of these exposures, which can be more harmful than the sum of their individual effects. The co-exposure paradigm acknowledges that environmental contaminants rarely exist in isolation; rather, individuals are exposed to multiple toxicants simultaneously, which may exert synergistic or antagonistic effects on biological systems. For instance, the Bayesian Kernel Machine Regression (BKMR) analysis used in a preliminary study on the association between combined metals and PFAS exposure with dietary patterns underscores the non-linear, non-additive relationships between PFASs and heavy metals in dietary inflammation, illustrating why a mixture-based approach is both necessary and timely [26]. Also, PFASs and heavy metals interact to influence lipid metabolism, revealing synergistic effects (e.g., mercury and PFASs jointly increasing cholesterol levels) and antagonistic effects (e.g., zinc potentially mitigating PFAS-induced oxidative stress) in a study on the associations of Per- and Polyfluoroalkyl substances and heavy metals with blood lipid profile [27]. The multipollutant/multiexposure approach provides a more accurate representation and helps identify the combined effects of various exposures [28].

This approach can help with public health decision-making, disaster management, and social services allocation, as some of these vulnerable populations are elderly individuals, low-income families, people with disabilities, and marginalized groups.

The objective of this study was to investigate the association between combined PFASs (PFOA and PFOS), metals (lead, cadmium, and mercury), social (income and education), and behavioral factors (smoking and alcohol consumption) on depressive symptoms. By investigating the combined effects of environmental, behavioral, and social factors, the study provides a more comprehensive understanding of how these factors collectively influence depression risk. By examining environmental, behavioral, and social factors, this study addresses critical gaps in the existing literature and deepens the understanding of depression’s multifaceted nature. We hypothesized that environmental, social, and behavioral factors would be jointly associated with depressive symptoms.

## 2. Materials and Methods

### 2.1. Study Design

This study employs a cross-sectional design utilizing data from the National Health and Nutrition Examination Survey (NHANES), a nationally representative survey of the U.S. population conducted by the Centers for Disease Control and Prevention (CDC). Data from cycles between 2017 and 2018 were analyzed, focusing on participants aged 20 years and older. The sample was selected based on the availability of data on PFASs (PFOA/PFOS), heavy metals (lead, cadmium, and mercury), behavioral factors (smoking and alcohol use), social factors (income and education), and depressive symptoms. The selection of specific PFAS compounds was driven by their documented prevalence in NHANES data, with PFOA and PFOS standing out as legacy chemicals extensively used in consumer goods and consequently the most comprehensively studied. Similarly, lead, cadmium, and mercury were chosen for their reliable detectability in biological samples, particularly among populations with distinctive exposure profiles.

### 2.2. Exposure Assessment

Blood samples were analyzed to determine concentrations of lead (Pb), cadmium (Cd), and total mercury (Hg) using mass spectrometry methods employed by NHANES. These metals were selected based on their known toxicity and potential impact on human health, particularly mental health outcomes. Blood lead levels were assessed as an indicator of both recent and cumulative exposure. Lead exposure occurs primarily through deteriorating lead-based paints, contaminated water, and occupational hazards. Lead in blood predominantly binds to red blood cells, and uniform distribution during analysis was ensured by mixing whole blood specimens. Blood cadmium reflects both recent and chronic exposures, primarily through inhalation (e.g., cigarette smoke) and ingestion (e.g., food sources). Smokers typically exhibit cadmium levels twice as high as non-smokers. Chronic exposure leads to accumulation in the liver and kidneys, contributing to long-term health effects. Total mercury in the blood was measured to account for exposure primarily from methylmercury in fish consumption and other sources. Vulnerable populations, including women of childbearing age, are particularly at risk of mercury-related health effects.

Laboratory analysis involved dilution of blood samples with a specific reagent mixture to release metals bound to red blood cells, followed by detection using inductively coupled plasma mass spectrometry (ICP-MS). Detection limits were set for each metal at 0.07 µg/dL, 0.10 µg/L, and 0.28 µg/L for lead, cadmium, and mercury, respectively. Analytical precision and accuracy were ensured through quality control measures, including blind split samples, repeat testing on 2% of specimens, and adherence to the Clinical Laboratory Improvement Amendments (CLIA) of 1988.

Perfluoroalkyl and polyfluoroalkyl substances (PFASs) are used in manufacturing polymers for industrial and consumer products like soil, stain, grease, and water-resistant coatings on textiles and carpets. These substances enter the human blood through ingestion, inhalation, skin contact, and occupational exposure. Quantitative detection of PFASs, n-perfluorooctane sulphonate (n-PFOS), and n-perfluorooctanoate (n-PFAO) was performed using online solid-phase extraction coupled with high-performance liquid chromatography and tandem mass spectrometry. This method separates, detects, and quantifies PFASs in serum with high sensitivity, achieving detection limits in the low parts per billion (ppb or ng/mL) range. The lower limit of detection (LLOD, in ng/mL) for both n-perfluorooctanoic acid (n-PFOA) and n-perfluorooctane sulfonic acid (n-PFOS) was 0.10. The reported results for all assays comply with the Division of Laboratory Sciences’ quality assurance and quality control (QA/QC) performance standards for accuracy and precision, aligning with principles similar to the Westgard rules.

### 2.3. Behavioral Factors

Smoking behavior was assessed using the NHANES variable, which measured the average number of cigarettes smoked per day during the past 30 days among participants who reported smoking. Smoking intensity was categorized into three groups for analysis: low intensity (1–10 cigarettes/day), moderate intensity (11–20 cigarettes/day), and high intensity (>20 cigarettes/day). Participants who did not report smoking were classified as “non-smokers”.

Alcohol consumption was assessed using the NHANES variable, which measured the frequency of heavy drinking episodes during the past 12 months. Heavy drinking was defined as consuming four or more drinks in a single day for females or five or more drinks for males. Participants were categorized into groups ranging from “non-drinkers” to “high-frequency drinkers.” NHANES behavioral data were collected using structured interviews with built-in consistency checks through the Computer-Assisted Personal Interview (CAPI) and Audio Computer-Assisted Self-Interview (ACASI) systems, along with standardized quality control procedures, to minimize recall and reporting biases in smoking and alcohol use data.

### 2.4. Social Factors

Income was assessed through NHANES categorical income variables, which grouped participants into brackets based on annual household earnings (e.g., USD 0–USD 4999, USD 5000–USD 9999, USD 10,000–USD 14,999, etc.). Education levels were self-reported by participants and classified into three categories: less than high school, high school graduate/GED, and some college or higher.

Trained interviewers used the Computer-Assisted Personal Interview (CAPI) system to conduct demographic questionnaires at home, allowing respondents to choose their interview language or request an interpreter. Hand cards with response choices were available in multiple languages, and interviewers guided respondents to the appropriate cards and provided additional assistance as needed.

### 2.5. Outcome Assessment: Depressive Symptoms

Depressive symptoms were evaluated using the Patient Health Questionnaire-9 (PHQ-9), a validated tool included in NHANES surveys. The PHQ-9 is a widely used screening tool for depression developed by Robert L. Spitzer, Janet B.W. Williams, and Kurt Kroenke in the late 1990s with funding from Pfizer [29]. It consists of nine items that assess symptom severity over the past two weeks. Responses are scored on a Likert scale ranging from 0 (not at all) to 3 (nearly every day). The total score is calculated by summing responses across all nine items, yielding a range of 0–27. Higher scores indicate more severe depressive symptoms. PHQ-9 scores were dichotomized into “no depression” (scores < 10) and “depression” (scores ≥ 10) based on established clinical thresholds.

### 2.6. Statistical Analysis

This study had complete data across all variables of interest; consequently, no missing data methods were necessary. All analyses were performed on a fully observed dataset without imputation or case-wise deletion.

#### 2.6.1. Descriptive Statistics

Summary statistics were calculated for all variables, including means and standard deviations for continuous variables (e.g., metal concentrations and PHQ-9 scores) and frequencies and percentages for categorical variables (e.g., smoking status and education levels).

#### 2.6.2. Spearman Correlation

Spearman’s correlation measures the strength and direction of a monotonic relationship between two variables using their ranked values instead of actual values. Spearman correlation was used to assess whether higher levels of PFAS or metals, combined with certain behavioral and social factors, consistently correspond to higher or lower incidence of depressive symptoms. Correlation coefficients (ρ) were calculated, and p-values were used to determine the statistical significance of the observed relationships. This methodology allows for a comprehensive analysis of the complex interactions between environmental exposures, behavioral and social factors, and their impact on depressive symptoms.

It is calculated based on the ranks of the data rather than the raw data values. The formula is as follows:

ρ = 1−6∑di^2^

       n(n^2^−1)

where:

- ρ is Spearman’s rank correlation coefficient;
- di is the difference between the ranks of corresponding values;
- *n* is the number of observations.

#### 2.6.3. Bayesian Kernel Machine Regression

To address the complexity of multipollutant exposures, Bayesian Kernel Machine Regression (BKMR) was used to estimate the individual and joint effects of PFASs and metals on depressive symptoms. These models account for potential correlations among exposures and allow for the identification of critical contributors. The BKMR method uses kernel functions to model the relationship between predictors and depressive symptoms. Given the complexity and potential non-linear relationships among these predictors, BKMR is an appropriate method to model these interactions and identify the most influential factors. BKMR was chosen for its ability to model complex, non-linear relationships and interactions among multiple predictors. The Gaussian kernel function was specified to capture these relationships. The hierarchical variable selection approach was implemented to identify important predictors while accounting for the correlated structure of the mixture. The BKMR model was fitted within a Bayesian framework. Prior distributions were specified for the model parameters, and Markov Chain Monte Carlo (MCMC) methods were used to estimate the posterior distributions. The multivariable exposure–response function, which captures the combined effects of the predictors on depressive symptoms, was estimated.

The BKMR model was represented by the following equation:

Y*i* = h(**X***i*) + Z*i*β + ϵ*_i_*

where:

- Y*i* is the outcome variable (depressive symptoms) for the *i*-th participant;
- h(X*i*) is a flexible, non-parametric function of the predictors X*i* (PFASs, metals, and behavioral and social factors);
- Z*i* represents additional covariates (e.g., age and gender) with corresponding coefficients β;
- ϵ*_i_* is the error term.

Visualization techniques, such as contour plots and interaction plots, were used to interpret these effects and the overall exposure–response function. Cross-validation was performed to assess the robustness of the model. The variable importance measures were interpreted to understand the relative contributions of each predictor to depressive symptoms. Higher values indicated stronger relative contributions. The overall effect of the mixture on depressive symptoms, as estimated using the BKMR model, was also interpreted. All models were adjusted for potential confounders, including age, gender, race/ethnicity, body mass index (BMI), and comorbid health conditions (e.g., diabetes, cardiovascular disease). In this study, data analysis was conducted using R (version 4.2.3; R Foundation for Statistical Computing, Vienna, Austria).

## 3. Results

### 3.1. Descriptive Statistics Results

Table 1 summarizes the descriptive statistics for the continuous variables measured in the study. The participant cohort (N = 181) was predominantly middle-aged (mean age ≈ 44 years) and had an average BMI near the obesity threshold, suggesting a population at increased risk of metabolic complications. The mean depression score (PHQ-9) indicated mild depressive symptoms on average. Notable differences were observed in PFAS exposure: PFOS concentrations were substantially higher than those of PFOA, suggesting differing environmental or biological accumulation patterns. Among metals, mercury exhibited greater variability compared to lead and cadmium, reflecting the presence of some participants with elevated levels likely due to differential exposure sources.

### 3.2. Spearman Correlation Analysis

**Figure 1:**
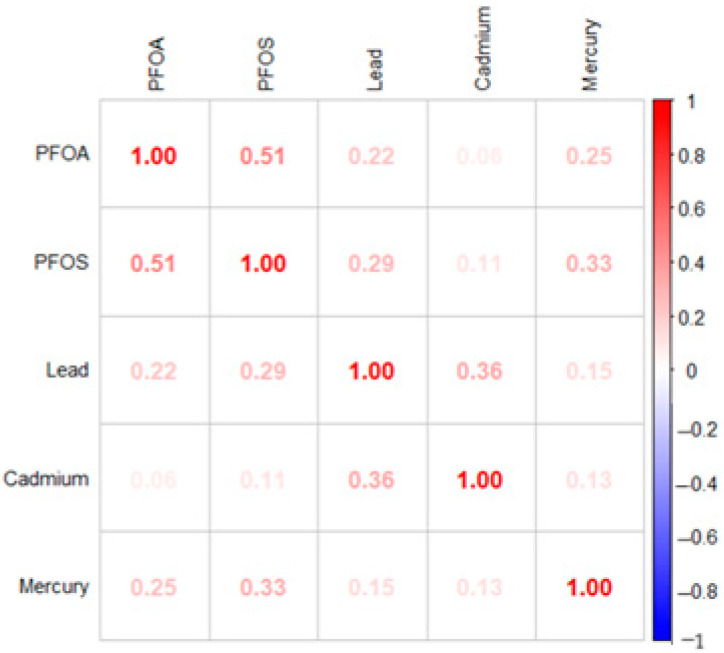
Spearman correlation matrix.

Figure 1 displays the Spearman correlation matrix for the key study variables, lead, cadmium, mercury, PFOA, and PFOS. Among the environmental contaminants, several significant positive correlations can be observed. PFOA and PFOS show the strongest correlation (0.51), indicating that these related perfluoroalkyl substances tend to co-occur in exposure scenarios. Lead demonstrates a moderate positive correlation with cadmium (0.36) and weaker positive associations with PFOS (0.29) and PFOA (0.22). Mercury correlates moderately with PFOS (0.33) and PFOA (0.25). These correlation patterns provide important context for understanding the complex interrelationships between multiple environmental exposures and depressive symptoms.

### 3.3. Logistic Regression

Table 2 presents the logistic regression analysis examining associations between depression and selected environmental, behavioral, and social factors. Lead was the only variable showing a statistically significant association with depression in the adjusted model. Mercury and education exhibited non-significant trends, while PFOA and cadmium showed small positive associations. Minimal associations were observed for PFOS, income, alcohol consumption, and smoking. All models were adjusted for age, gender, BMI, and ethnicity.

### 3.4. Bayesian Kernel Machine Regression Analysis

#### 3.4.1. Posterior Inclusion Probability Analysis

Table 3 presents the Bayesian Kernel Machine Regression results, highlighting the group and conditional posterior inclusion probabilities for environmental, behavioral, and social factors associated with depressive symptoms. The group PIP highlights the overall importance of environmental variables as a collective, assessing their combined influence on the model. In contrast, the conditional PIP isolates the impact of individual variables, determining their significance within the group. The environmental variables, lead, cadmium, and mercury, are grouped together with a group PIP of 0.6508, indicating their collective importance in the model. Among them, cadmium has the highest conditional PIP (0.3509), suggesting a stronger individual contribution compared to lead (0.3146) and mercury (0.3343). Social factors, including income and education, are grouped with a group PIP of 0.6420. Within this group, education exhibits a higher conditional PIP (0.5862) compared to income (0.4137), indicating education’s greater individual contribution to depressive symptoms in the study. Behavioral factors, including alcohol consumption and smoking, are grouped with a group PIP of 0.5508. Within this group, alcohol exhibits a higher conditional PIP (0.5047) compared to smoking (0.4952), indicating alcohol’s greater individual contribution to depressive symptoms in this study.

#### 3.4.2. Univariate Exposure Response

**Figure 2:**
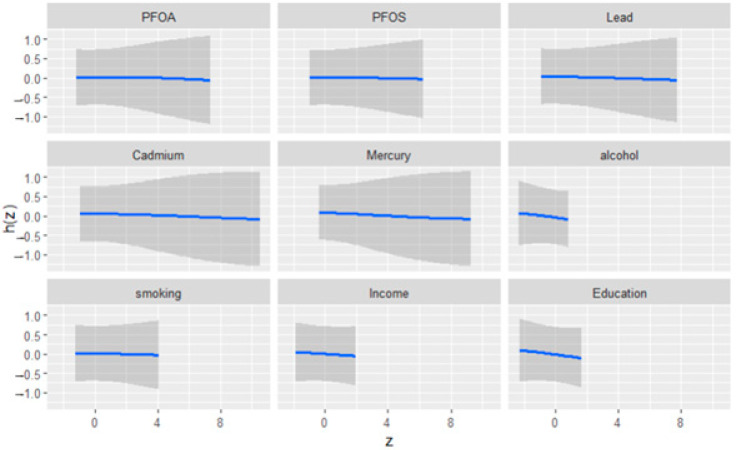
Univariate exposure effect curves of the relationship between individual predictor variables and depression levels while all other predictors were held at their median. The blue line represents the posterior mean estimate, and the gray area indicates the 95% credible interval.

Figure 2 illustrates the univariate exposure–response functions and their 95% credible intervals for the association between environmental exposures (PFOA, PFOS, lead, cadmium, and mercury), social factors (income and education), behavioral factors (alcohol consumption and smoking), and depressive symptoms. The figure demonstrates how each exposure relates to depression when controlling for other variables in the model. The exposure–response curves reveal consistent patterns across all variables, indicating limited univariate effects on depressive symptoms.

#### 3.4.3. Bivariate Exposure Response

**Figure 3:**
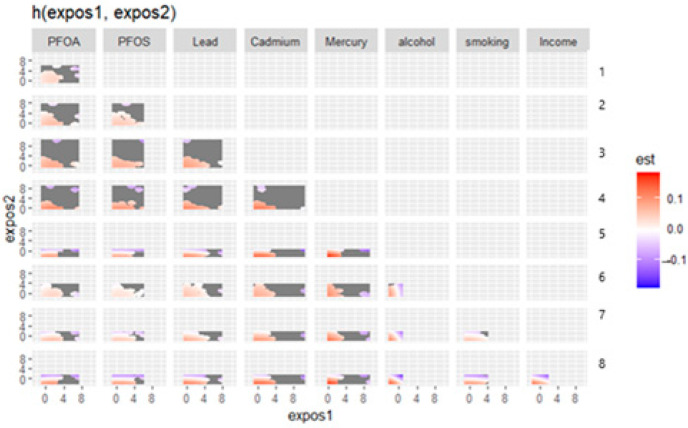
Bivariate exposure response. Right axis: 1—PFOS, 2—Lead, 3—Cadmium, 4—Mercury, 5—Alcohol, 6—Smoking, 7—Income, and 8—Education.

Figure 3 displays the bivariate exposure–response functions for pairs of metals, behavioral factors, and social factors in relation to depressive symptoms. This complex plot visualizes the interaction effects between pairs of environmental exposures, behavioral factors, and social variables on depressive symptoms. Each cell represents how two factors jointly influence depression risk, with color indicating effect direction and magnitude (red = increased risk; blue = protective effect). Bivariate exposure–response analysis revealed complex interaction patterns between environmental contaminants. The effect of each exposure on depressive symptoms varied considerably depending on co-exposure levels, with certain combinations showing protective effects and others showing increased risk.

#### 3.4.4. Overall Effect Summary

**Figure 4:**
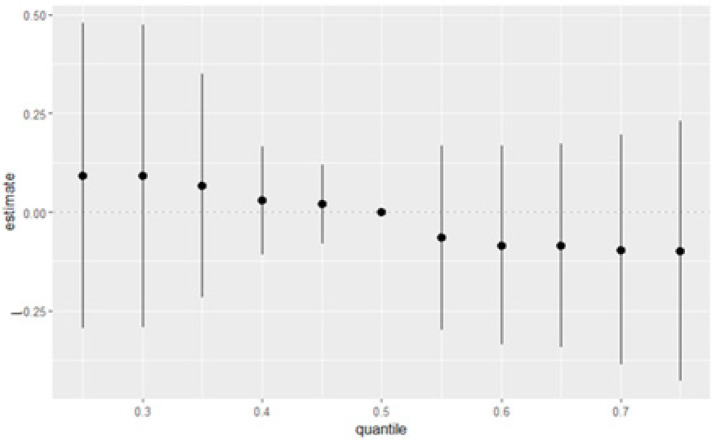
Overall effects of the eight variables with a 95% credible interval. This figure shows the estimated changes in depression when the eight variables were set at particular percentiles (ranging from 25th to 75th percentiles) compared to when all variables were at their 50th percentile. Black dots represent estimates with lines representing 95% credible intervals.

Figure 4 presents the summary of overall exposure effects on depressive symptoms, analyzed across different quantiles, ranging from the 25th to 75th percentiles. The *y*-axis represents the estimated effect on depression, while the *x*-axis shows the quantiles of exposure to all of the exposure variables. Each point in the plot represents the effect estimate at a specific quantile, with the vertical lines indicating the 95% credible intervals around these estimates. The figure demonstrates a clear trend in how exposure effects vary across the depression distribution. At lower quantiles (0.25–0.35), representing individuals with milder depressive symptoms, the estimated effects are positive, suggesting potential depression risk-increasing relationships. As we move toward the median (0.5 quantile), the effect estimates approach zero, indicating minimal impact. At higher quantiles (0.55–0.75), representing individuals with more combined exposure, the estimated effects become increasingly negative, suggesting less depression.

#### 3.4.5. Single-Exposure Effect Analysis

**Figure 5:**
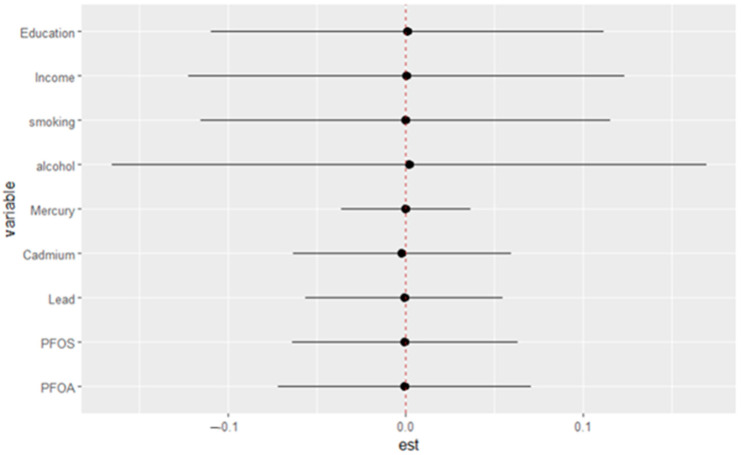
Single-variable interaction terms. Each estimate reflects how the effect of an exposure (from the 25th to 75th percentile) differs when other exposures are fixed at the 25th versus the 75th percentile.

Figure 5 illustrates the estimated effects of various exposures on depressive symptoms, with each horizontal line representing the 95% credible interval for the corresponding variable. The point estimates (black dots) indicate the magnitude and direction of each exposure’s association with depressive symptoms, while the vertical red dotted line at zero represents the null effect. The exposures/variables examined include social factors (education and income), behavioral factors (smoking and alcohol), and environmental contaminants (mercury, cadmium, lead, PFOS, and PFOA). This visualization offers a comprehensive comparison of each exposure’s effect by evaluating the impact of shifting from the 25th to the 75th percentile under two conditions: first, when all other exposures are held constant at the 25th percentile, and second, when all others are held at the 75th percentile. The difference between these two estimated effects is the interaction contrast, which captures how the effect of an individual exposure varies depending on the background levels of the other exposures.

**Figure 6:**
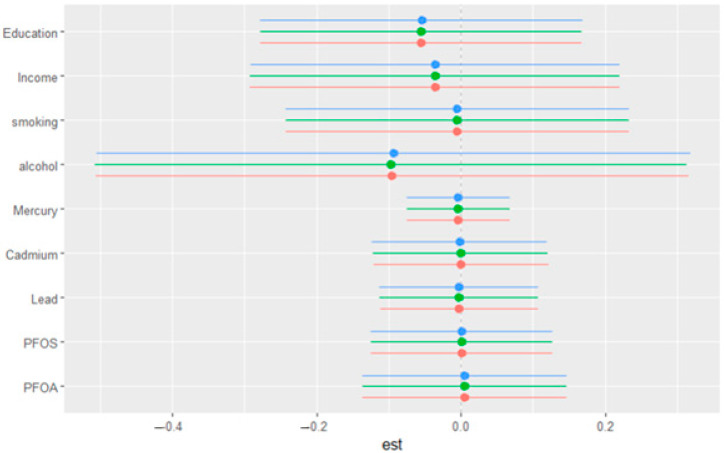
Single-variable effect. Plot showing the changes in depression levels with a 95% credible interval in a single variable when all other variables were fixed at either the 25th, 50th, or 75th percentile.

Figure 6 illustrates the single-exposure/variable effects on depressive symptoms, showing the estimated changes in the response when a particular exposure shifts from its 25th to its 75th percentile, with all other exposures/variables held constant at different quantiles (0.25, 0.50, or 0.75). The exposures/variables included are education, income, smoking, alcohol consumption, mercury, cadmium, lead, PFOS, and PFOA. The horizontal lines represent the 95% confidence intervals for each estimate, with different colored points corresponding to the three quantiles (blue for 0.25, green for 0.50, and red for 0.75). The plot provides a comparison of how the effect of each exposure/variable on depressive symptoms varies depending on the quantile at which other exposures are held. This figure offers an in-depth examination of how individual exposures interact with depressive symptoms across different levels of depression severity, emphasizing that exposure effects appear relatively stable regardless of where in the depression distribution an individual falls.

## 4. Discussion

This study aimed to investigate the combined effects of PFASs (PFOA and PFOS), metals (lead, cadmium, and mercury), behavioral (alcohol and smoking), and social factors (income and education) on depressive symptoms. The results revealed unexpected patterns in the relationship between environmental exposures and depression. The observed protective association between lead exposure and the outcome (OR = 0.38) in the logistic regression model appears paradoxical, especially given the well-established neurotoxic effects of lead. However, this counterintuitive finding likely reflects limitations inherent in the frequentist framework and the logistic regression model, particularly when dealing with complex, potentially non-linear relationships and correlated exposures [30,31,32].

First, logistic regression assumes a linear relationship between the log-odds of the outcome and the predictors. This assumption may not hold in environmental health contexts, where the dose–response relationship between toxicants like lead and health outcomes is often non-linear and may involve thresholds, plateaus, or even U-shaped curves. If these complexities are not adequately modeled, the regression may yield misleading estimates.

Second, logistic regression struggles to account for interactions and high collinearity among covariates without explicitly modeling them. Lead exposure is often intertwined with various social and environmental stressors (e.g., lower socioeconomic status, poor housing, and limited healthcare access), which may confound or modify its effect. Inadequate control or mis-specification of these relationships can obscure the true direction and magnitude of the association.

Moreover, the frequentist approach typically provides point estimates and confidence intervals without fully capturing uncertainty due to model selection, potential unmeasured confounding, or data sparsity. In small or imbalanced datasets, or when covariates are highly correlated, this can lead to unstable estimates or even reversal of expected effects—sometimes referred to as “statistical paradoxes”. In contrast, social factors such as education may demonstrate more consistent protective associations because their relationships with health outcomes are often more direct and linear, and they are less likely to be subject to the same complex interactions or measurement challenges as environmental exposures [33,34,35]. Altogether, this underscores the need for more flexible and robust modeling approaches—such as Bayesian Kernel Machine Regression—that can better accommodate non-linearities, interactions, and uncertainty in exposome data [36].

The observed discrepancies in these results arise from differences in the underlying assumptions and methodological flexibility of both models. While logistic regression imposes a linear assumption, BKMR provides greater adaptability, allowing it to capture non-linear and interactive effects that simpler models might overlook. This distinction is particularly important, as BKMR is specifically designed to evaluate mixture effects, offering a more nuanced perspective on complex exposure–outcome relationships that may not be fully captured through logistic regression analysis.

Alcohol’s relationship with depression exhibited substantial uncertainty, and most environmental exposures showed no significant associations when examined individually. These findings suggest that the complex interactions between multiple exposures likely play a more important role than individual contaminant effects, highlighting the limitations of traditional single-exposure approaches in environmental mental health research.

In a study published by Aung et al., the authors found that exposure to per- and polyfluoroalkyl substances, including PFOS, was associated with a higher incidence of depressive symptoms among immigrant women during pregnancy. This suggests a potential negative impact of PFOS on mental health in this demographic [37]. Conversely, in a study by Tang et al. on “unraveling the link between heavy metals, perfluoroalkyl substances and depression: insights from epidemiological and bioinformatics strategies” [38], the authors found that PFOS exposure was negatively associated with depression, indicating a potential protective effect. However, the authors noted that further research is needed to understand this relationship fully.

Heavy metals collectively showed the strongest evidence for influencing depressive symptoms, with the highest group posterior inclusion probability (0.6508), with cadmium having the highest conditional importance within this group. Social factors demonstrated the second highest group probability (0.642), with education showing the highest conditional inclusion probability (0.586) of any individual variable across all groups. PFAS chemicals and behavioral factors show moderate group probabilities (0.554 and 0.551, respectively) with balanced within-group importance, suggesting that these exposures play a role in depressive symptoms but with less certainty than heavy metals and social factors.

The relationship between environmental exposures and mental health outcomes involves intricate interaction patterns that go beyond simple linear associations. The bivariate exposure–response plot gave deeper insights into the relationships between two exposures and depressive symptoms.

This analysis revealed a complex interplay between various environmental contaminants, behavioral factors, and social variables in relation to depressive symptoms, highlighting the multifaceted nature of environmental health effects.

The bivariate exposure–response plots generated from the BKMR model indicated that, across most combinations of exposures, increased levels of depression were observed. These results suggest that the joint effects of environmental toxicants, behavioral factors, and social determinants tend to compound risk rather than operate independently [39]. Notably, pairs such as PFOS and lead, as well as lead and cadmium, showed clear positive gradients across quadrants of their respective plots, indicating that individuals with varying levels of exposure to both factors simultaneously had the great predicted levels of depression. This pattern supports the hypothesis that the cumulative burden of chemical exposures—particularly neurotoxicants and endocrine-disrupting compounds—can lead to heightened vulnerability to depressive symptoms, especially when experienced in tandem. Also, PFASs and heavy metals disrupt biological pathways linked to depression through oxidative stress, neuroinflammation, neurotransmitter dysregulation, endocrine disruption, and blood–brain barrier permeability. While heavy metals contribute to oxidative damage and neuroinflammation, PFASs interfere with hormonal balance and lipid metabolism, creating potential synergistic effects that amplify neurotoxicity [40,41].

Additionally, behavioral factors such as alcohol consumption and smoking demonstrated meaningful interactions with chemical exposures. The combination of high smoking or alcohol use with elevated levels of cadmium or mercury was associated with stronger depressive responses, reinforcing the notion that behavioral and environmental stressors interact synergistically to exacerbate mental health outcomes. Importantly, these associations appeared to follow a graded response pattern, rather than a threshold effect, suggesting that even moderate levels of joint exposure may incrementally elevate the risk of depression. This aligns with the concept of allostatic load, whereby the cumulative wear and tear from multiple sources of stress—whether chemical, behavioral, or psychosocial—contributes to declining mental health.

Collectively, these findings point toward a multi-exposure level interaction model in which chemical exposures, health behaviors, and social determinants jointly influence depression risk. The results underscore the relevance of the external exposome in shaping mental health outcomes. Moreover, they suggest that interventions aiming to mitigate depression risk should consider both environmental remediation and structural supports, such as education and income enhancement, to effectively address the broader determinants of mental well-being. Additionally, the complex interaction patterns demonstrate that environmental influences on mental health cannot be adequately understood through single-exposure approaches. The effect of any given contaminant on depressive symptoms appears highly contingent upon co-exposures to other chemicals, lifestyle factors, and social factors.

The overall exposure effect plot reveals a nuanced, non-linear association between the combined exposure mixture and depression. Interestingly, lower quantiles of the exposure mixture—representing individuals with relatively modest levels of cumulative exposure—appear to show a slight positive association with depression, whereas higher quantiles demonstrate a subtle decline in the estimated effect. Although these trends are uncertain given the wide credible intervals, the pattern suggests that lower levels of combined exposures may be more strongly associated with depression than higher levels. This counterintuitive finding is critical, as it implies that the dose of exposure may not linearly explain the psychological burden of the full mixture. Rather, it points toward a more complex dynamic in which moderate levels of diverse stressors may more acutely impact emotional well-being.

One possible explanation lies in the concept of cumulative burden overload. Individuals at the highest quantiles of exposure may be subject to such extensive and persistent stress—across environmental, behavioral, and socioeconomic domains—that depression is no longer the predominant or most measurable manifestation of distress. In such cases, the burden may shift toward physical deterioration, toxicological damage, or other adverse outcomes, such as cardiovascular disease or immune dysregulation. Conversely, those at lower or moderate levels of the mixture may not be chronically exposed to such a breadth of stressors, and the convergence of multiple exposures—even at relatively low doses—could represent a novel or acute challenge to their psychological resilience. As a result, depression may surface more prominently among those who are not habituated to chronic environmental or psychosocial stress, reflecting a form of vulnerability rooted in relative exposure novelty rather than total toxic load. These findings underscore the importance of moving beyond dose–response linearity in mixture research and recognizing how exposure context, individual resilience, and social conditioning intersect to shape mental health outcomes.

Overall, this study provides a unique multivariable assessment of the combined effects of multiple exposures. By leveraging BKMR to capture both individual and interactive effects within a complex mixture, this work contributes valuable insights into how depression symptoms may emerge from real-world exposure profiles. These findings highlight the need for public health approaches that move beyond single-exposure frameworks and instead address the intersectionality of environmental, behavioral, and structural determinants in shaping mental health outcomes.

From a public health perspective, traditional approaches often focus on single-exposure risks, such as individual toxicants or behavioral factors. However, this study reinforces the need for multi-exposure frameworks that consider cumulative effects and interactions between pollutants, social determinants, and lifestyle behaviors. Effective public health strategies should integrate environmental regulations, such as reducing exposure to PFASs and heavy metals, with mental health initiatives that provide accessible counseling, preventive care, and social support systems. Policymakers could focus on reducing environmental inequities by targeting populations disproportionately exposed to neurotoxicants, such as low-income communities and occupationally at-risk groups. Targeted policies supporting socioeconomic advancement may help reduce the psychological stressors that intensify mental health risks. From a clinical standpoint, healthcare providers should consider environmental and socioeconomic exposures when evaluating mental health conditions. Screening tools like the PHQ-9 could be supplemented with assessments of environmental toxicant burdens and social risk factors, providing a more holistic approach to patient care. Additionally, therapeutic interventions could integrate nutritional, behavioral, and pharmacological strategies to address both biological and socio-environmental contributors to depression.

### Limitations

Several limitations should be acknowledged. First, the cross-sectional design of the study limits the ability to infer temporality or establish causal relationships between exposure mixtures and depressive symptoms as measured using the PHQ-9. Without longitudinal data, it remains unclear whether the exposures preceded the onset of depression or if individuals with depressive symptoms are more likely to encounter certain exposures or adopt specific behaviors. Second, although PHQ-9 is a validated tool for assessing depressive symptoms, it captures self-reported symptoms over a short time frame and may not reflect clinical diagnoses or long-term mental health trajectories. Additionally, unmeasured confounding—particularly related to early-life exposures, genetic susceptibility, diet, or psychosocial stressors—may influence the observed associations.

## 5. Conclusions

This study demonstrates that depressive symptoms are shaped by a complex interplay of environmental, behavioral, and social exposures. While individual contaminants showed limited associations with depression, Bayesian Kernel Machine Regression revealed that joint exposure patterns—particularly involving heavy metals and social factors like education—play a more significant role. The findings underscore the importance of adopting multi-exposure modeling frameworks in environmental mental health research and highlight the need for integrated public health interventions that consider both environmental remediation and social determinants to effectively mitigate depression risk. As such, these findings are best understood as exploratory, offering initial insights that merit subsequent investigation through more extensive, longitudinal research frameworks.

## Figures and Tables

**Table 1:** Descriptive statistics for continuous variables.

Variable	N	Mean	St. Dev
PHQ	181	4.856	5.378
PFOA	181	1.605	1.250
PFOS	181	5.598	5.605
Lead	181	1.463	1.449
Cadmium	181	1.138	1.132
Mercury	181	1.129	2.466
Age	181	44.315	14.892
BMI	181	29.546	8.307

**Table 2:** Logistic regression results for depression.

Variable	* Odd Ratio	St. Dev	Pr (>|z|)	95% Conf. Interval
Lead	0.38	1.449	0.0425	0.12947650, 0.8368766
Cadmium	0.59	1.132	0.5355	0.92999428, 1.2871442
Mercury	1.16	2.466	0.156	0.24902967, 1.0545558
PFOA	1.18	1.250	0.5614	0.65401879, 1.9597077
PFOS	0.96	5.605	0.5461	0.81721292, 1.0969616
alcohol	1.09	3.127	0.3124	0.02011353, 18.5627477
smoking	0.99	7.317	0.7639	0.91878889, 1.0585707
Education	0.68	1.014	0.0961	0.42594223, 1.0671882
Income	0.91	2.871	0.2443	0.76994398, 1.0649660

* Adjusted for age, gender, BMI, and ethnicity.

**Table 3:** BKMR analysis of depressive symptoms: group and conditional posterior inclusion probabilities.

Variable	Group	Group PIP	Cond PIP
PFOA	1	0.5536	0.5253
PFOS	1	0.5536	0.4747
Lead	2	0.6508	0.3147
Cadmium	2	0.6508	0.3510
Mercury	2	0.6508	0.3344
Alcohol	3	0.5508	0.5047
Smoking	3	0.5508	0.4953
Income	4	0.6420	0.4137
Education	4	0.6420	0.5863

## Data Availability

The data presented in this study are openly available on the CDC NHANES site at https://wwwn.cdc.gov/nchs/nhanes/continuousnhanes/overview.aspx?BeginYear=2017 (accessed on 1 April 2025).
